# A Prospective Clinical Study on Blood Mercury Levels Following Endodontic Root-end Surgery with Amalgam

**Published:** 2013-08-01

**Authors:** Masoud Saatchi, Elham Shadmehr, Seyed Morteza Talebi, Mohsen Nazeri

**Affiliations:** a Torabinejad Dental Research Center, Department of Endodontics, Dental School, Isfahan University of Medical Sciences, Isfahan, Iran; b Department of Chemistry, Isfahan University, Isfahan, Iran; c Private Practice, Isfahan, Iran

**Keywords:** Dental Amalgam, Endodontics, Mercury, Root Canal Filling Materials, Oral, Surgery

## Abstract

**Introduction:**

The purpose of this clinical study was to compare the blood mercury levels before and after endodontic surgery using amalgam as a root-end filling material.

**Materials and Methods:**

Fourteen patients requiring periradicular surgery participated in this prospective clinical study. A zinc-free amalgam was employed as root-end filling material. Blood samples were collected at three intervals: immediately before, immediately after and one week postoperatively. Mercury content of the blood was determined using gold amalgamation cold-vapor atomic absorption spectrometry. Obtained data were analyzed using analysis of variance for repeated measures and paired t-test.

**Results:**

The mean (SD) of blood mercury levels was 2.20 (0.24) ng/mL immediately before surgery, 2.24 (0.28) ng/mL immediately after surgery and 2.44 (0.17) ng/mL one week after the periradicular surgery. The blood mercury level one week post-operative was significantly higher than both blood mercury levels immediately before (*P<0.001*) and immediately after (*P=0.005*) the surgery.

**Conclusion:**

Placement of an amalgam retroseal during endodontic surgery can increase blood mercury levels after one week. The mercury levels however, are still lower than the toxic mercury levels. We suggest using more suitable and biocompatible root-end filling materials.

## Introduction

Periradicular surgery is an important adjunct to orthograde root canal therapy. When non-surgical treatment fails or cannot be performed, surgical approach is indicated [1, 2]. Apical surgery usually consists of periapical curettage followed by root-end resection, cavity preparation, and filling. A retrograde filling material is usually used to seal the root-end cavity and prevent microleakage. A good quality root-end filling is essential for a successful endodontic surgery [3].

The ideal root-end filling material should be biocompatible, bioinductive, bactericidal or at least bacteriostatic, insoluble in tissue fluids, dimensionally stable, easy to use, radiopaque, non-toxic, non-carcinogenic, and not corrosive. It should also be electrochemically inactive, and non-staining. It should have excellent sealing ability and promote regeneration of the original tissues [4]. Although, the ideal material is yet to be found, a number of materials have been suggested for root-end filling including amalgam, composite resin, glass ionomer cement, gold foil, gutta-percha, reinforced zinc oxide eugenol based cement, mineral trioxide aggregate (MTA), and calcium enriched mixture (CEM) cement [5-8].

Amalgam has been a frequently used root-end filling material; it is easy to use, radiopaque and non-resorbable [9]. The characteristics of amalgam as a root-end filling material such as marginal adaptation [10], sealing ability [11], cytotoxicity [12] and biocompatibility [5] have been evaluated. Some concerns have been expressed regarding the release of mercury from amalgam into the bloodstream [13]. Mercury is the most harmful of all heavy metals; moreover, can change the distribution and retention of other heavy metals [14]. It is a very reactive metal that has many recognized toxic properties at high doses including cerebellar ataxia, paresthesia, dysarthria, and constriction of the visual fields [15]. Mercury may also be a risk factor in multiple sclerosis [16] and Alzheimer's disease [17].

In spite of studies that evaluate the release of mercury ions from amalgam restorations into the blood or urine [18-20], there have been few studies evaluating the blood mercury levels following amalgam root-end filling materials [21, 22]. Since amalgam retroseals have a direct contact with periradicular tissue fluids, they may release mercury in a different pattern compared with amalgam restorations. In this prospective clinical study, we aimed to evaluate the blood mercury levels immediately before, immediately after, and one week following the placement of freshly mixed amalgam as a root-end filling material using gold amalgamation cold-vapor atomic absorption spectrometry method (GA-CVAAS).

## Material and Methods

The study consisted of 15 patients who were referred for surgical endodontic treatment to the Department of Endodontics, School of Dentistry, Isfahan University of Medical Sciences, Iran. The Ethics Committee of the University approved the protocol of the study. Written informed consent was obtained from all patients. Patients who were older than 18 years with no physician-diagnosed immunosuppressive, neurological, psychological, behavioral, or renal disorders, and had a tooth requiring periradicular surgery were included in the study. Subjects who are exposed to mercury in their job like dentists, stonemason, and mine workers were not included in the study. Patients who used alcohol and cigarette or tobacco chewing habit during the study, ate seafood one week before and during the study, took medications that might affect mercury assessment, were excluded from the study. Patients were all required to provide informed consent if they were to take part in the study. One patient was excluded due to the reasons above.

### Surgical procedure

The teeth were treated by a senior specialist. Root-end resection was performed with an International Organization for Standardization (ISO) #14 sterile tapered fissure bur (Maillefer, Ballaigues, Switzerland) using a straight handpiece and sterile saline coolant. Approximately, 3 mm of the root-end was resected as close to 90° to the long axis of the root. Root-end preparation was performed using ultrasonically powered tips numbers E31D or E32D (NSK Varios 750, Nakanishi, Tochigi, Japan). Approximately, a 3-mm deep cavity was prepared. In all the surgical treatments zinc free amalgam (Tytin Kerr Sybron, Romulus, MI, USA) was used. For each subject, blood samples were collected in three intervals of immediately before and immediately after the surgery, and also one week later. In each interval 10 mL blood was drawn and coded using single blind protocol.

### Mercury analysis

The blood samples were mixed with 0.5 mL of 1% EDTA (Merck, Darmstadt, Germany) anticoagulant agent and kept frozen at -20°C. A method based on GA-CVAAS was used for the determination of trace mercury (Hg+2) in the blood samples. This method was developed for ultra-trace mercury determination [23, 24]. Determinations were done on a Shimadzu model AA-6601F single beam atomic absorption spectrometer (Nakagyo-Ku, Kyoto, Japan) and calculated as nanogram per milliliter (ng/mL).

### Statistical Analysis

The data were analyzed using SPSS software, version 15 (SPSS Inc, Chicago, IL). Analysis of variance for repeated measures was used to compare the blood mercury levels of patients at three intervals, followed by paired t-test. Statistical significance was defined at P<0.05.

## Results

One patient was excluded as he did not have the necessary criteria. Of the 14 patients selected in our study, 8 were men and 6 were women. Their age ranged from 27-56 years with a mean age of 40 years. The individual and the mean (standard deviation) blood mercury levels of patients at three intervals are shown in Table 1. There was no significant difference between the total blood mercury levels immediately before and immediately after the surgery (P=0.315). One week after surgery, the total mercury concentration in the blood was significantly higher than both immediately before (P<0.001) and immediately after the surgery (P=0.005). 

**Table 1.** The minimum, maximum, mean (SD) of blood mercury levels of subjects at three intervals (ng/mL)

Interval	Min.	Max.	Mean (SD)
Immediately before the surgery	1.8	2.6	2.2 (0.24)
Immediately after the surgery	1.9	2.7	2.24 (0.28)
One week after the surgery	2.1	2.7	2.44 (0.17)

## Discussion

Mercury can be found in three basic forms of elemental, inorganic, and organic. Dental amalgams are one of the most common sources of elemental mercury. Amalgam mercury is methylated to organic mercury in the oral cavity and/or gastro-intestinal tract [25, 26]. Fish and sea mammals are the sources of organic mercury in the form of methyl and ethyl mercury. Inorganic mercury is the toxic species found in human tissue after conversion from the other forms [27]. The investigations about the effects of mercury content of dental amalgam are still ongoing [28].

Dental amalgam is composed of 50% mercury, 25% silver, 25% tin, copper, and nickel. It has been the main source of human exposure to mercury [26, 28]. Also, amalgam has historically been the most widely used root-end filling material for more than a century [29]. It is economical and easy to manipulate. However, it has several disadvantages including corrosion, electrolysis, delayed expansion, marginal leakage, and causing tissue tattoos [30, 31]. Mercury toxicity has been a further deterrent to the selection of amalgam as a restoration and/or root-end filling material [32].

In our study, a significant elevation of blood mercury levels was seen in the patients following the placement of amalgam root-end fillings. Our results do not coincide with previous studies [21, 22]. Longos et al. reported baseline blood and urine mercury levels for 10 female baboons that underwent root end surgery with amalgam. They assessed amalgam levels using cold vapor atomic absorption spectrometry method (CVAAS) [21]. Blood and urine samples were monitored at the time of surgery and at 24 hours, 48 hours, 1 week, 2 weeks, 1 month, and 2 months after surgery. They found that mercury was undetectable in the majority of samples. In a few samples, they found barely detectable levels of mercury. They concluded that mercury releases from retrograde amalgam fillings is of little concern. Skoner et al. analyzed blood mercury level of 10 patients requiring endodontic surgery using an amalgam retroseal [22]. They measured blood mercury levels using CVAAS at four intervals of one week before surgery, at the time of surgery, one week after surgery, and one month after surgery. They reported that placement of amalgam retroseals did not increase the blood mercury levels significantly. The difference between the findings of our study and two other studies may be because of the using different techniques for detection of blood mercury levels. Although the CVAAS method is effective, popular, and widely accepted for the determination of mercury in biomedical samples, the GA-CVAAS method offers a lower detection limit and high sensitivity [24, 33] therefore we used GA-CVAAS method which is more accurate. The method is able to determine the ultra trace amount of mercury. Also, we did not include the patients who smoked or consumed alcohol. Tobacco smoke can increase the absorption of mercury because cigarette smoking may be a substantial source of intake of hazardous elements such as mercury [34]. Alcohol depresses oxidation and retention of mercury in most organs and whole body and thus increase blood level of mercury [35].

The mean blood mercury level was 2.44±0.17 ng/mL at one week after the surgery which was significantly more than the mean blood mercury levels immediately before and immediately after the surgery. The normal blood mercury level is considered to be in the range of 0-5 ng/mL. Toxic blood mercury level is reported to be 200 ng/mL and the lethal level to be 600 ng/mL [22, 36]. In our study the maximum blood mercury level was 2.7 ng/mL; this value is within normal range. However, the release of mercury from amalgam retroseal may continue and potentially threaten the health of individuals. Therefore, we suggest using more suitable root-end filling materials [8, 37]. Because the results of our study show increased blood mercury level at one week after the surgery, we also recommend long-term studies on this subject.

A total of 20 mL blood was drawn for each patient in 2 intervals of immediately before and immediately after the surgery. To prevent blood pressure related problems, only healthy individuals should be selected.

## Conclusion

In conclusion, based on the results of this clinical study, mercury is released from amalgam after endodontic surgery as a root-end filling material. Although the amount of mercury released is smaller than the toxic mercury levels, amalgam retroseals may release this heavy metal over time.
